# The relationship between parenting attitudes, negative cognition, and the depressive symptoms according to gender in Korean adolescents

**DOI:** 10.1186/s13033-016-0069-3

**Published:** 2016-04-27

**Authors:** Subin Park, Bung-Nyun Kim, Min-Hyeon Park

**Affiliations:** Research Planning Division, Mental Health Research Institute, National Center for Mental Health, 127, Yongmasan-ro, Gwangin-gu, Seoul, 04933 Republic of Korea; Department of Psychiatry and Behavioral Science, Seoul National University College of Medicine, Seoul, Republic of Korea; Department of Psychiatry, The Catholic University of Korea, Seoul St. Mary׳s Hospital, Seoul, Republic of Korea

**Keywords:** Parenting, Depression, Negative cognition, Adolescents

## Abstract

**Background:**

Parenting style is one potential contributor to the development of adolescents’ cognitions, self-esteem and emotional problems. This study examined the relationship between maternal parenting attitudes and adolescents’ negative cognitions, and depressive symptoms according to gender.

**Methods:**

A total of 401 middle and high school students were recruited (i.e. 221 males and 180 females; mean age, 13.92 ± 1.31 years). The Maternal Behavior Research Instrument assessed maternal parenting attitudes. Analyses examined the relationship between parenting attitudes and affective symptoms, with self-esteem and negative automatic thoughts as mediators of these relations.

**Results:**

Maternal rejecting attitudes were positively associated with depressive symptoms via increasing negative autonomic thoughts and decreasing self-esteem among female adolescents. Among male adolescents, maternal rejecting attitudes were associated with low self-esteem, but they were not associated with depressive symptoms.

**Conclusions:**

Maternal parenting has a larger impact on the emotional adjustment of females compared to males. Interventions to increase self-esteem and correct negative cognitions may be helpful for depressed female adolescents, specifically for those whose mothers are rejecting.

## Background

Many adolescents suffer from emotional problems including depression. Indeed, lifetime prevalence rates of depression range from 7 to 15 % [1–3]. Depressive symptoms have been found to have detrimental impacts on adolescent social and emotional development. Research has shown that parenting behaviors (e.g., support and control) are correlated with adolescent depressive symptoms and problem behavior [4, 5] In the late 1950s, Schaefer et al. [6] proposed two independent dimensions that were important for understanding parenting styles. One dimension involves parents’ emotional or affectionate attitudes toward the child (i.e. affection vs. rejection); the other dimension reflects parents’ exerting control over the child’s behavior (i.e. autonomy vs. control). Four parenting styles (i.e. authoritative, authoritarian, permissive, and neglecting) have been derived from these two dimensions [7, 8]. Authoritative parenting combines high support and flexible control, and may have the most benefits for children and adolescents as it is related to several positive outcomes, including self-esteem and emotional adjustment, as well as low aggression, anxiety, and depression [9–11]. In contrast, authoritarian parenting is high in rigid control and lacks support, permissive parenting lacks control, and neglecting parenting lacks support and control. These styles are related to negative outcomes for children and adolescents [11, 12].

Most studies on parenting style and it’s relation to depression have not examined the role of child gender; however, there are some indications in the literature that parenting style may have a more significant influence on psychological outcomes for daughters than for sons. For college women, but not college men, permissive mothering was positively related to stress, anxiety, and depression, while authoritative mothering was negatively related to anxiety and depression [13]. For female adolescents, perceived authoritative and permissive parenting was positively related to self-esteem and negatively related to depression and anxiety, while authoritarian parenting had opposite effects. In contrast, for male adolescents, authoritative and permissive parenting was not related to emotional adjustment, but authoritarian parenting was positively related to anxiety [14].

One way that parenting behavior might contribute to adolescent depressive symptoms and problem behavior is through adolescent cognitive vulnerability. Negative thought patterns and maladaptive information processing-termed “cognitive vulnerabilities”-have been shown to contribute to the development of depressive symptoms in adolescents [15, 16]. Cognitive vulnerability theories (i.e., Beck’s cognitive theory [17], hopelessness theory [18], and the cognitive vulnerability-transactional stress model [19]) hypothesize that individuals who possess cognitive vulnerabilities are more likely to develop depressive symptoms after the occurrence of a negative event than are individuals who do not possess such vulnerabilities. These negative cognitions cause biased interpretations of negative events, resulting in overly negative views of the self, world, and future, which in turn promote the development of depressive symptoms. Based on our understanding of the cognitive vulnerability factors for depression, evidence that a maladaptive parenting style prospectively predicts these cognitive vulnerabilities would support the potential role of cognitive vulnerabilities as a mechanism linking parenting and offspring depression. Two prospective studies have indicated that negative parenting styles predict a subsequent cognitive vulnerability in offspring. In a sample of 6th grade children, Garber and Flynn [20] found that maternal affectionless control assessed at Time 1 predicted children’s lower self-worth 1 year later. Similarly, Koestner et al. [21] found that parenting style assessed when children were 5 years old predicted their levels of self-criticism at age 12. Specifically, maternal rejection predicted subsequent self-criticism among daughters, whereas paternal rejection predicted self-criticism among sons.

Recent research has suggested that the gender discrepancy in depressive disorders (e.g., higher rates of depressive disorder in females) may be at least partially accounted for by differences between women and men in cognitive patterns of negative feelings. According to the cognitive vulnerability-transactional stress theory of depression [19], women’s responses to negative events are characterized by rumination and a negative inferential style, which are often associated with depression, to a greater degree than are men’s. Hanking and Abramson [16] found that cognitive characteristics, such as negative inferences about the self, mediated gender differences in depressive symptoms.

As an extension of the above model, we examined whether male and female adolescents differ in their responses to negative parenting styles, such as by adopting a negative cognitive style, which in turn may be associated with differing likelihoods of developing depression. Given that available research has shown that girls exhibit a more negative cognitive style than do boys [19] and girls are more influenced by parenting style than are boys [13, 14], the association between parenting style and offspring depression via cognitive vulnerability may be more prominent among girls than among boys. This study attempts to clarify the complex relationship between maternal parenting attitudes and adolescents’ cognitive vulnerability, and depressive symptoms by gender. Based on previous research on parenting behavior’s influence on self-esteem and cognition, which are often associated with depressive symptoms, we hypothesized that (1) parenting attitudes are related to depressive symptoms, specifically for female adolescents and (2) this relationship is mediated by cognition and self-esteem.

## Methods

### Participants, ethics, consent, and permissions

A total of 646 students between 7th and 10th grade (age range: 12–15 years old) were recruited from one junior high school and one senior high school that were located in Seoul, South Korea. These schools were volunteered to participate in this study. After the school principals approved the research, the investigators visited the schools, explained the purpose of the study to the students and teachers, obtained consent, and distributed and collected the questionnaires (i.e., the children’s automatic thought scale, the Rosenberg self-esteem scale, and the children’s depression inventory). The authors also mailed letters to parents that outlined the study’s objectives, guaranteed confidentiality, provided a contact telephone number for the principal investigator and indicated that parents would be informed of the results after the analyses were completed. The letter also included a statement that parents were free to refuse to respond if they did not agree with the study’s objective. Mothers were asked to complete questionnaires about parenting attitudes (the maternal behavior research instrument), which were returned after 3 days. This study was approved by the human subjects institutional review board at Seoul National University Hospital. A total of 401 parents responded to the letters and completed the questionnaires (221 boys, 180 girls). Participants mean ± standard deviation (SD) age was 13.92 ± 1.31 years.

### Measures

#### The children’s automatic thought scale (CATS)

The CAT is a self-report measure that assesses a wide range of negative self-statements in children and adolescents. Four separate cognitive content subscales are assessed, including physical threat (e.g., I’m going to get hurt), social threat (e.g., I’m worried that I’m going to get teased), personal failure (e.g., I can’t do anything right), and hostility (e.g., I have the right to take revenge on people if they deserve it) (Cronbach’s alpha = 0.83, 0.92, 0.90, and 0.75, respectively). Each subscale has 10 items and is scored by summing the subscale responses (rated on a scale between 0 and 4). Subscale scores ranged from 0 to 40. The total score is the sum for all subscales (range 0–160) [22, 23]. The CATS is designed for children and adolescents between 8 and 17 years old.

#### The Rosenberg self-esteem scale (RSES)

The RSES is a widely used self-report instrument that measures global self-worth. It has ten items (e.g., I feel that I have a number of good qualities, Cronbach’s alpha = 0.80). Participants answer each item on a 5-point Likert scale, ranging from 1 (strongly disagree) to 5 (strongly agree). The total score ranges from 10 to 50 [24, 25].

#### The children’s depression inventory (CDI)

The children’s depression inventory (CDI) is a self-rated, symptom-oriented scale that is suitable for youth aged 7 to 17. There are 27 items quantifying symptoms such as depressed mood, hedonic capacity, vegetative functions, self-evaluation, and interpersonal behaviors (Cronbach’s alpha = 0.82). Each CDI item consists of three statements that are rated on a scale that ranges from 0 to 2. The total score ranges from 0 to 54 [26, 27].

#### The maternal behavior research instrument (MBRI)

The MBRI is a 48-item, self-report instrument that assesses maternal parenting attitudes. Parenting attitudes are classified into affective (e.g., “It is fun to spend time with my child,” Cronbach’s alpha = 0.80), rejecting (e.g., “I ignore my child’s demands,” Cronbach’s alpha = 0.76), autonomic (e.g., “I let my child do his/her own thing alone,” Cronbach’s alpha = 0.70), and controlling (e.g., “Children should by all means be obedient to their parents,” Cronbach’s alpha = 0.67). Each parenting attitude has 12 items and is scored by summing the subscale responses (values range between 1 and 5). Subscale scores range between 12 and 60. Higher scores on each subscale indicate that maternal parenting attitudes corresponds to a greater degree to that attitude [6, 28].

### Statistical analyses

Descriptive statistics were used to describe the basic features of the data in a study. Pearson correlation analyses were conducted to determine the correlation between variables. Next, we tested our hypothesized meditational model. We used Baron and Kenny’s traditional method [29] and confirmed the findings with the more rigorous Sobel statistical tests [30]. Two models explored mediation by negative automatic thoughts or low self-esteem on the relationship between maternal parenting attitudes and adolescents’ depressive symptoms. Then, we conducted a multigroup structural equation modeling (SEM) analysis using AMOS (version 20.0; SPSS Inc., Chicago, IL) to examine the possible moderating effect of gender—namely, that female adolescents would be more likely to show greater negative automatic thoughts with stronger maternal rejecting attitudes, and to report more severe depressive symptoms with greater negative automatic thoughts compared to male adolescents. These moderating effects were estimated using Chi square difference tests. Model fit was examined with the Adjusted Goodness of Fit Index (AGFI), Non-Normed Fit Index (NNFI), and Root Mean Square Error of Approximation (RMSEA). All statistical analyses were performed using SPSS software (version 21.0; SPSS Inc., Chicago, IL), with statistical significance defined as an alpha level < 0.05.

## Results

Table 1 shows mean, skewness, and kurtosis of the variables among the total, female, and male samples.

**Table 1:** Characteristics of study participants

	Total (N = 401)			Girls (N = 228)			Boys (N = 221)		
	Mean (SD)	Skewness (SE)	Kurtosis (SE)	Mean (SD)	Skewness (SE)	Kurtosis (SE)	Mean (SD)	Skewness (SE)	Kurtosis (SE)
Affective attitudes	43.41 (5.80)	−0.50 (0.12)	1.54 (0.24)	43.19 (5.71)	−0. 85 (0.18)	3.66 (0.36)	43.58 (5.89)	−0. 24 (0.16)	0.01 (0.33)
Rejecting attitudes	31.37 (5.85)	−0.06 (0.12)	0.33 (0.24)	31.07 (5.96)	0.04 (0.18)	−0. 07 (0.36)	31.61 (5.77)	−0. 13 (0.16)	0.77 (0.33)
Autonomic attitudes	41.49 (5.29)	−0.60 (0.12)	2.45 (0.24)	41.83 (5.30)	−0. 49 (0.18)	0.99 (0.36)	41.21 (5.27)	−0. 71 (0.16)	3.74 (0.33)
Controlling attitudes	41.35 (5.46)	0.07 (0.12)	0.19 (0.24)	41.89 (5.43)	−0. 09 (0.18)	0.60 (0.36)	40.90 (5.46)	0.19 (0.16)	−0.002 (0.33)
CATS	21.38 (24.44)	1.60 (0.12)	2.15 (0.24)	21.79 (24.61)	1.72 (0.18)	2.62 (0.36)	21.05 (24.36)	1.52 (0.16)	1.85 (0.33)
RSES	29.79 (6.14)	0.09 (0.12)	−0.45 (0.24)	29.17 (5.93)	0.01 (0.18)	−0.34 (0.36)	30.29 (6.28)	0.11 (0.16)	−0.57 (0.33)
CDI	14.57 (7.17)	0.33 (0.12)	−0.35 (0.24)	15.86 (6.85)	0.22 (0.18)	−0. 59 (0.36)	13.52 (7.26)	0.47 (0.16)	−0.05 (0.33)

*CATS* children’s autonomic thought scale, *RSES* *The Rosenberg’s self*-*esteem scale*, *CDI* children’s depression inventory

For female adolescents, maternal rejecting attitudes were negatively correlated with self-esteem (r = −0.22, p = 0.003) and positively correlated with negative autonomic automatic thoughts (r = 0.21, p = 0.004) and depressive symptoms (r = 0.19, p = 0.012). For male adolescents, self-esteem was negatively and positively correlated with maternal rejecting attitudes (r = −0.18, p = 0.008) were negatively and maternal affective attitudes (r = 0.20, p = 0.002), respectively were positively correlated with self-esteem (r = −0.18, p = 0.008 and r = 0.20, p = 0.002). There were no significant correlations between any parenting attitudes and depressive symptoms in male adolescents. For both female and male adolescents, depressive symptoms were self-esteem was negatively and positively correlated with self-esteem (r = −0.37, p < 0.001 and r = −0.26, p < 0.001) and negative autonomic automatic thoughts were positively (r = 0.46, p < 0.001 and r = 0.36, p < 0.001), respectively) correlated with depressive symptoms (Table 2).

**Table 2:** Correlations between parenting attitudes, cognitive variables, and depressive symptoms by gender

	Female						Male					
	Affective attitudes	Rejecting attitudes	Autonomic attitudes	Controlling attitudes	CATS	RSES	Affective attitudes	Rejecting attitudes	Autonomic attitudes	Controlling attitudes	CATS	RSES
Affective attitudes												
Rejecting attitudes	−0.28**						−0.43**					
Autonomic attitudes	0.47**	−0.15*					0.49**	−0.17*				
Controlling attitudes	0.29**	0.29**	−0.01				0.29**	0.14*	0.20			
CATS	−0.08	0.21**	0.01	0.03			−0.01	0.01	−0.02	−0.01		
RSES	0.10	−0.22**	0.09	−0.07	−0.52**		0.20**	−0.18**	0.09	0.11	−0.32**	
CDI	−0.08	0.19*	−0.07	−0.03	0.46**	−0.37**	−0.17	0.10	−0.11	−0.02	0.36**	−0.26**

*CATS* children’s autonomic thought scale, *RSES* *The Rosenberg’s self-esteem scale*, *CDI* Children’s depression inventory

* p < 0.05, ** p < 0.01

Hierarchical regression analyses indicated that when negative automatic thoughts were added as a mediating variable between rejecting attitudes and female adolescents’ depression, the relationship between parenting style and depression became non-significant (model 1). Likewise, when self-esteem was added as a mediator, the relationships between the mother’s rejecting attitudes and the daughters’ depression became non-significant (model 2) (Table 3). Sobel tests for both models indicated that negative automatic thoughts or self-esteem were full mediators in the association between maternal rejecting attitudes and depressive symptoms (Z = 2.66, p = 0.004 for model 1 and Z = 2.59, p = 0.005 for model 2).

**Table 3:** Mediation in the relationship between maternal rejecting attitudes and female adolescents’ depressive symptoms

Model		Step1		Step2		Step3	
		Autonomic thoughts (model 1) or self-esteem (model 2)		Depressive symptoms		Depressive symptoms	
		B (SE)	p	B (SE)	p	B (SE)	p
1	Rejecting attitudes	0.87 (0.30)	0.004**	0.22 (0.09)	0.012*	0.11 (0.08)	0.173
	Autonomic thoughts					0.12 (0.02)	<0.001**
	F	8.34	0.004**	6.43	0.012*	25.31	<0.001**
2	Rejecting attitudes	−0.22	0.003**	0.22 (0.09)	0.012*	0.13 (0.08)	0.118
	Self-esteem	(0.07)				−0.40 (0.08)	<0.001**
	F	8.87	0.003^**^	6.43	0.012^*^	15.38	<0.001**

Step 1 includes maternal rejecting attitudes as independent variables with the hypothesized mediator (autonomic thoughts or self-esteem) as a dependent variable

Step 2 includes maternal rejecting attitudes as independent variables with depressive symptoms as a dependent variable

Step 3 includes maternal rejecting attitudes and the hypothesized mediator (autonomic thoughts or self-esteem) as dependent variables with depressive symptoms as a dependent variable

Baron and Kenny’s method. (* p < 0.05, ** p < 0.01)

**Fig. 1:**
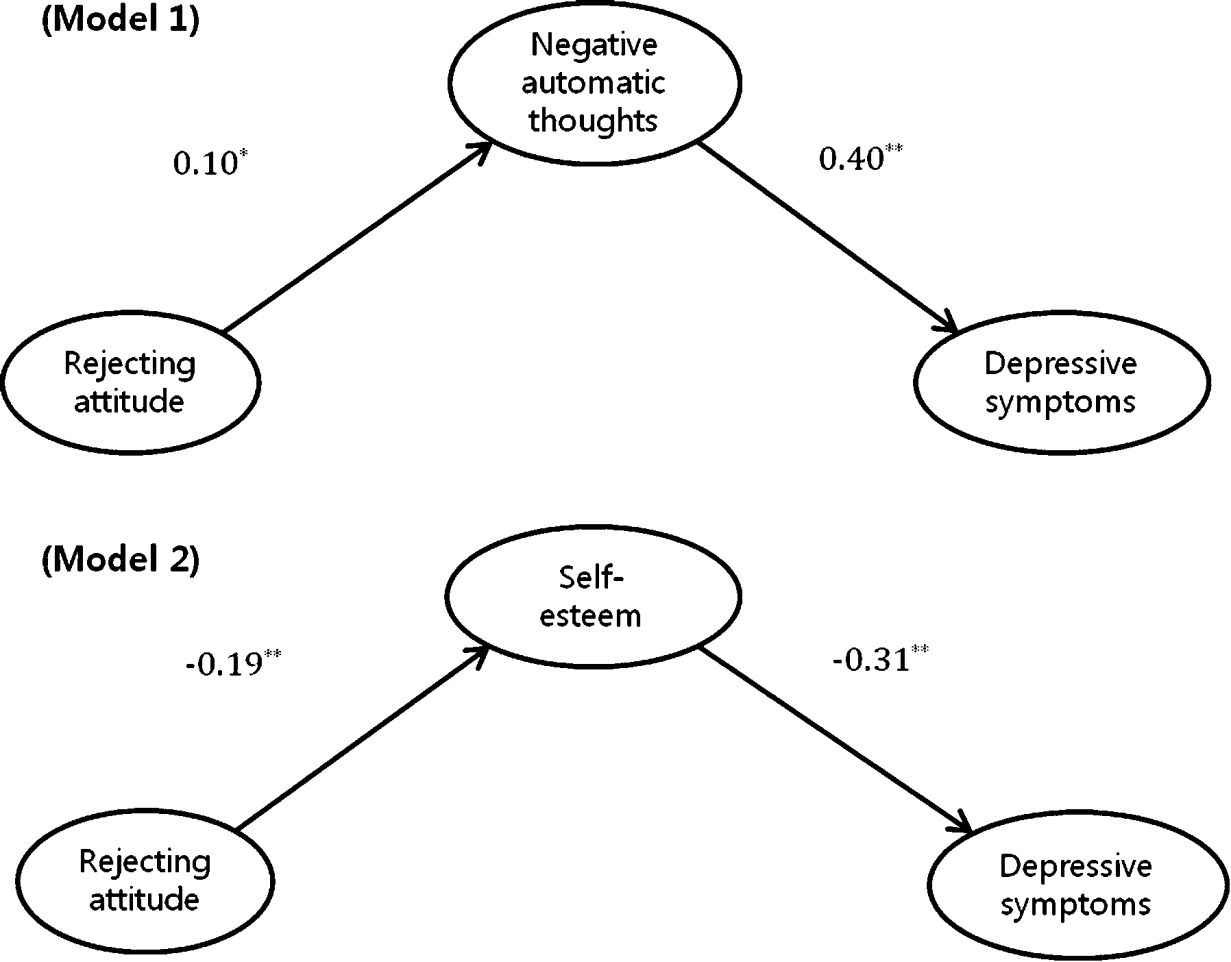
Structural equation modeling (SEM) results in the total sample and standardized path coefficients. The relationship between maternal rejecting attitudes and depressive symptoms was fully mediated by negative automatic thoughts (*model 1*) or self-esteem (*model 2*). Only significant paths are presented (*p < 0.05, **p < 0.001)

Figure 1 shows the results from the SEM analysis for the total sample, which indicated that rejecting parenting attitudes predicted high depressive symptoms through more negative automatic thoughts (model 1) or low self-esteem (model 2). In model 1, the path of rejecting parenting attitudes to negative automatic thoughts (β = 0.10, p = 0.037) was significant, as was the path from negative automatic thoughts to depressive symptoms (β = 0.40, p < 0.001). The fit indices indicated that the model had a good fit to the data (AGFI = 0.96, NNFI = 0.91, RMSEA = 0.05). In model 2, the path of rejecting parenting attitudes to self-esteem (β = −0.19, p < 0.001) was significant, as was the path from self-esteem to depressive symptoms (β = −0.31, p < 0.001). The fit indices indicated that the model had a good fit to the data (AGFI = 0.98, NNFI = 0.96, RMSEA = 0.03).

**Fig. 2:**
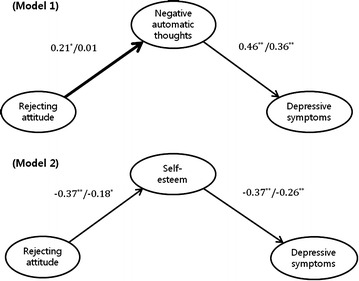
Multigroup structural equation modeling (SEM) results and standardized path coefficients. The *bold line* indicates a pathway that is significantly different between female and male adolescents. Only significant paths are presented (* p < 0.01, ** p < 0.001)

Figure 2 shows the results from the multigroup SEM analysis for male and female adolescents. The structural models for male and female adolescents were compared in two different pathways. First, male and female groups were compared by freeing the path from maternal parenting attitudes to negative automatic thoughts (model 1) or self-esteem (model 2), after which the $\chi^{2}$ for this model with one parameter freed was compared with the $\chi^{2}$ of a fully restrained model. Second, male and female adolescents were compared by successively freeing the paths from negative automatic thoughts or self-esteem to depressive symptoms; the same $\chi^{2}$ comparison procedures were then followed. In model 1, for female adolescents, the path of rejecting parenting attitudes to negative automatic thoughts (ß = 0.21, p = 0.004) was significant, as was the path from negative automatic thoughts to depressive symptoms (ß = 0.46, p < 0.001). In contrast, for male adolescents, the path of rejecting parenting attitudes to negative automatic thoughts (ß = 0.01, p = 0.832) was not significant, whereas the path from negative automatic thoughts to depressive symptoms was significant (ß = 0.36, p < 0.001). Thus, the results of the multigroup SEM analysis of model 1 indicated a moderating effect of gender on the association between maternal rejecting attitudes and negative automatic thoughts ($\Delta \chi^{2}$ (1) = 3.85 > $\chi^{2}$ 0.05(1) = 3.84), although there was no moderating effect of gender on the association between negative automatic thoughts and depressive symptoms ($\Delta \chi^{2}$ (1) = 0.78). In model 2, for both female and male adolescents, the path of maternal rejecting attitudes to self-esteem (ß = −0.37, p < 0.001 and ß = −0.18, p = 0.007, respectively) was significant, as was the path from self-esteem to depressive symptoms (ß = −0.37, p < 0.001 and ß = −0.26, p < 0.001, respectively). Thus, the results of the multigroup SEM analysis of model 2 did not indicate any moderating effect of gender on the association between maternal rejecting attitudes and self-esteem ($\Delta \chi^{2}$ (1) = 0.05) or between self-esteem and depressive symptoms ($\Delta \chi^{2}$ (1) = 1.36).

## Discussion

Our results revealed that mother’s rejecting attitudes were significantly associated with depressive symptoms in female adolescents, and this association was fully mediated by negative automatic thoughts and low self-esteem in those adolescents. For male adolescents, mother’s rejecting attitudes were significantly associated with low self-esteem, but not with depressive symptoms.

The associations between less affective and more rejecting parenting attitudes and adolescents’ psychological outcomes are consistent with previous studies [11–13]. Consistent with previous research [11, 13], we found stronger relationships between maternal parenting attitudes and emotional outcomes for female adolescents compared to male adolescents. These results suggest that female adolescents may be more susceptible than males to maternal parenting attitudes. An additional explanation is that mothers may adopt different parenting behaviors based on their adolescents’ gender (e.g., more involvement in daughters’ parenting than in sons’), which may be related to affective outcomes. Another consideration is that only maternal parenting attitudes—and not paternal attitudes—were investigated in the present study. Some researchers have suggested that the parenting style of the same-sex parent is more related to depression in the offspring than is the style of the opposite-sex parent. Indeed, in their meta-analysis, Gerlsma et al. [31] reported larger effect sizes for same-sex parents. Although the reason that same-sex parenting may have a stronger effect than opposite-sex parenting is unclear, it is possibly related to the child showing greater identification with the same-sex parent [18].

A considerable amount of research has demonstrated that negative automatic thoughts are a major factor associated with depression in the adolescent population [32] and that they mediate the effect of negative life events and maladaptive parenting on depression [33]. Jackson et al. [9] found that optimism significantly mediated the negative relationship between authoritativeness and depression. McKinney et al. [11] found that students’ positive and negative perceptions significantly altered the relationships between parenting style and depression. Researchers have also suggested that inadequate parenting such as parental rejection and coercion may lead adolescents to negatively evaluate themselves and their future (e.g., low self-esteem), which, in turn, might make adolescents vulnerable to depressive symptoms [34]. On the other hand, positive parenting behaviors, such as support and monitoring, might contribute to adolescents’ positive views of self, which can in turn protect them from developing internalizing symptoms [34]. However, these studies did not examine whether there are gender differences in the relationships between parenting and adolescent depression via maladaptive cognitions or self-esteem. Adding to the existing research, we found that negative automatic thoughts and low self-esteem fully mediate the relationships between maternal rejecting parenting and depressive symptoms for female adolescents. Specifically, maternal rejecting attitudes predicted negative automatic thoughts in female adolescents, but not in male adolescents. These results support the cognitive models that hypothesize gender differences in cognitive patterns surrounding negative feelings, such as the cognitive vulnerability-transactional stress theory of depression [19].

Our findings suggest a number of future directions for researching the ways in which negative automatic thoughts or low self-esteem may be alleviated to help prevent female adolescents of rejecting mothers from experiencing depressive symptoms. Changing negative cognitions and raising self-esteem are important for preventing and treating depression Strong relational and communal networks are important relational factors for teenagers who have high self-esteem. Thus, providing social supports from peers and mentors may have protective benefits for female adolescents who have rejecting mothers. Cognitive behavioral therapy to modify negative thoughts and beliefs may also be helpful for these adolescents. For male adolescents, we could not test for mediation because no relationship was detected between parenting attitudes and depressive symptoms.

Our study had several limitations. First, this study was cross-sectional, thus, we could not identify causal relationships between parenting attitudes, automatic thoughts, self-esteem, and depressive symptoms. Second, only maternal parenting attitudes were investigated in the present study, although there is a large body of evidence to show that the behaviors of both parents influence child’s psychological outcomes [35–37] and the primary caregiver in some families may not be the mother but may instead be the father or grandparents. Third, additional factors, such as stressful life events and personality traits, were not accounted for in this study. These factors co-vary with parenting attitudes and may be pathways to negative cognitions, low self-esteem, and depressive symptoms. Third, the diagnosis of depression by clinical and/or structured assessments was not possible in this study. Finally, the schools volunteered to participate in the study. Therefore, our findings may not be representative of all Korean adolescents. Future prospective studies with more representative samples and with additional measures, including paternal parenting attitudes, are needed.

## Conclusion

This study extended findings from previous research by examining the mediating effect of negative cognitions and low self-esteem on the relationship between parenting attitudes and depressive symptoms by gender. One clinical implication of this study is that treatment for adolescents who have emotional problems should include detailed family assessments that assess parenting styles and interventions to raise self-esteem and correct negative cognitions, specifically for female adolescents who have depressive symptoms.

## Abbreviations

CATS: the children’s automatic thought scale
RSES: the rosenberg self-esteem scale
CDI: the children’s depression inventory
MBRI: the maternal behavior research instrument
